# Procedural sedative effect of remimazolam in ICU patients on invasive mechanical ventilation: a randomised, prospective study

**DOI:** 10.1186/s13613-025-01431-5

**Published:** 2025-01-14

**Authors:** Youli Tian, Jintong Li, Minggen Jin, YiHua Piao, Jisheng Sheng, Zhixiong Mei, Qingsong Cui, Lilin Li

**Affiliations:** 1 Department of Intensive Care Unit, Yanbian University Hospital, No. 1327, Juzi Street, Xinxing Street, Yanji, 136200 Jilin China; 2 Department of Intensive Care Unit, Linyi People’s Hospital, Shandong, 276000 China

**Keywords:** Remimazolam, Propofol, Analgesia and sedation, Intensive care unit

## Abstract

**Background:**

Invasive procedures and environmental factors in the intensive care unit (ICU) may cause anxiety and discomfort in patients, who often require sedation therapy. The aim of this study was to assess the safety of remimazolam tosilate for procedural sedation in ICU patients receiving mechanical ventilation following endotracheal intubation. Eighty patients from a single centre were randomly assigned to either the propofol group or the remimazolam group. Blood tests were conducted to evaluate changes in lactate, blood lipids, liver and kidney function, and inflammatory markers, and patients’ vital signs were observed over several periods. This study compared the incidence of delirium, the impact on liver and kidney function, circulatory effects, and changes in blood lipids between the two groups. These findings have optimised the selection of medications, providing ICU patients with more options for sedation therapy.

**Methods:**

In this single-centre randomised controlled trial, intubated patients were randomly assigned to the remimazolam group or the propofol group. Under the same analgesic regimen, the two groups received remimazolam and propofol for procedural sedation.

**Results:**

Our primary outcome was the mean arterial pressure (MAP), which significantly differed on Days 4 and 7 (*P* = 0.021, control group vs. experimental group = 85.23 ± 11.24 vs. 94.36 ± 13.18, *P* = 0.023, 83.55 ± 8.94 vs. 92.66 ± 7.02). With respect to liver and kidney function, the ∆AST value in the remimazolam group was significantly lower than that in the control group on Day 7 (*P* = 0.023). There were significant differences in triglyceride (TG) levels on Days 4 and 7 (*P* = 0.020) and in the ∆LDL on Day 7 (*P* = 0.027). Furthermore, the rates of dyslipidaemia and delirium in the remimazolam group were lower than those in the propofol group (85.0%, n = 40 vs. 90.0%, n = 40; 27.5%, n = 40 vs. 55%, n = 40).

**Conclusion:**

Remimazolam is a novel benzodiazepine that has demonstrated promising applications in general anaesthesia and procedural sedation; however, its use in ICU sedation is still in the early stages of research. Current evidence suggests that remimazolam is a safe sedative that is particularly well suited for patients with haemodynamic instability. Large sample-size randomised clinical trials are warranted.

**Supplementary Information:**

The online version contains supplementary material available at 10.1186/s13613-025-01431-5.

## Introduction

With the development of critical care medicine, analgesia and sedation therapy have become indispensable for ICU patients. Proper sedation and analgesia can relieve tension and anxiety in patients, reduce pain and discomfort, reduce stress responses, prevent adverse states in patients from interfering with treatment, improve sleep, protect life, and help with disease management. Propofol and midazolam are the most commonly used traditional sedative medications. Numerous studies have examined the efficacy of both drugs in critically ill patients [1, 2]. Propofol has a rapid onset of action, and patients recover quickly after discontinuation [3, 4]. However, propofol can cause adverse effects such as respiratory depression and hypotension [5]. In addition, it is an ester drug that can cause lipids to accumulate in the body, leading to dyslipidaemia [6]. Although midazolam has a lesser effect on circulatory suppression, it is a lipophilic drug with a long half-life, and its active metabolites can lead to prolonged sedation time, consequently extending the duration of mechanical ventilation and hospital stay [7]. Therefore, a new sedative to treat these patients is urgently needed. Remimazolam is a derivative of midazolam that achieves a sedative effect by inhibiting the firing of neurons in the substantia nigra reticular structure of the brain [8]. The drug has a rapid onset of action, rapid recovery, and is not metabolised by the liver or kidneys but is metabolised by carboxylesterase in tissue [9] and does not accumulate in vivo [10]. Remimazolam has relatively stable haemodynamic properties and poses a lower risk of hypotension [6]. In their study on the effective dosage and safety of remimazolam in ICU patients undergoing invasive mechanical ventilation, Chen et al. reported that a loading dose of 0.02–0.05 mg/kg followed by a maintenance dose of 0.20–0.35 mg/kg/h could achieve a satisfactory postoperative sedation effect [11]. In a study of remimazolam for long-term sedation in patients on invasive mechanical ventilation in the ICU, remimazolam was noted to be effective and safe [12]. In addition, the authors of the study noted in a separate article that remimazolam, which is used for short-term deep sedation in critically ill patients, is a safe and effective drug [13]. Therefore, remimazolam is an ideal drug for patients with severe disease. At present, research on the effects of remimazolam on blood lipids, liver and kidney function, haemodynamics, and the incidence of delirium is lacking. This study compared various aspects, including haemodynamic stability, liver and kidney function, the incidence of delirium, and treatment costs, of remimazolam with the conventional sedative propofol. The objective of this study was to verify the safety of the use of remimazolam tosilate for procedural sedation in ICU patients receiving mechanical ventilation following endotracheal intubation.

## Methods

This single-centre, randomised, controlled, single-blind, prospective study was approved by the Ethics Committee of the Affiliated Hospital of Yanbian University (2,021,151). This clinical study was registered at ClinicalTrials.gov (ChiCTR2400091004).

### Patients

The inclusion criteria for this study were patients in the intensive care unit (ICU) receiving endotracheal intubation and invasive mechanical ventilation who provided informed consent either personally or through their families.

The exclusion criteria were as follows: (a) age ≤ 18 years and > 75 years; (b) patients who underwent surgery; (c) pregnancy or lactation; (d) severe, preexisting parenchymal liver disease with clinically significant portal hypertension; Child–Pugh C grade liver cirrhosis; or acute liver failure; (e) bronchial asthma or myasthenia gravis; f. history of alcohol or drug abuse; (g) any condition that impedes proper assessment of cognitive function, such as language and sensory disorders or mental disorders (language difficulties or organic mental disorders); (h) BMI < 18.5 kg/m^2^ or > 35 kg/m^2^; (i) history of the use of monoamine oxidase inhibitors, such as phenylethylhydrazine and isoniazid, within 14 days; (j) inability to obtain informed consent or authorization; (k) patient participation in other exploratory clinical trials within 6 months before screening; and (l) allergy to the experimental drugs.

**Fig. 1:**
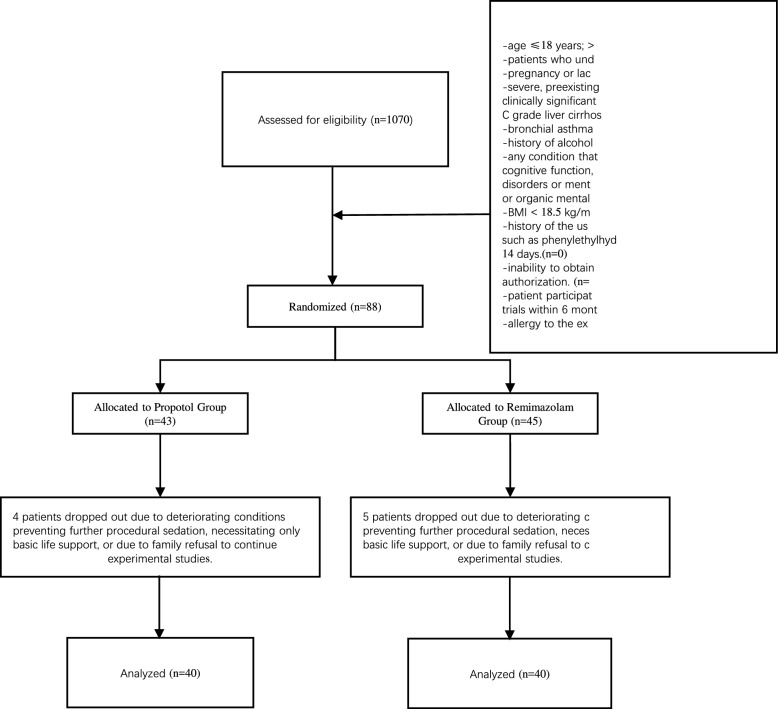
Flow chart of the study

We recruited 1070 patients who underwent endotracheal intubation and mechanical ventilation from December 2021 to December 2022. Among them, 88 patients who received mechanical ventilation and met the inclusion criteria were chosen to participate in this study. However, eight patients were excluded from the study because of clinical deterioration, which rendered them unable to continue sedation and analgesia treatment. These patients were instead provided with basic life support, or further experimental intervention was declined by their families (Fig. 1).

### Randomization and intervention

The critically ill patients who met the inclusion criteria were randomly divided into two groups via both the random number table method and the random number remainder grouping method. The critically ill patients included in this study were numbered, starting from any number in the random number table, and each patient was obtained in the same direction in order. The random number was divided by the number of groups (this study was divided into 2 groups) to find the remainder and to group the patients according to it. The patients were randomly divided into two groups: the propofol sedation group (control group) and the remimazolam besylate sedation group (experimental group). The researchers and research nurses involved in the study were aware of the grouping and medication used. Blinding was applied to the participating patients so that they would not know their own research drugs.

Daily wake-up was carried out at 08:30–09:00 in the morning, and the Confusion Assessment Method of Intensive Care Unit (CAM-ICU) rating scale was used to evaluate whether the patient experienced delirium. The detailed scoring criteria are presented in Supplementary Table 1. During this research, we consistently monitored vital signs. Changes in the patient’s vital signs on the 0th, 1st, 4th, and 7th days were observed. Indicators such as lactate (Lac), liver function, kidney function, blood lipids, and inflammation were monitored by testing blood samples. Statistical methods were used to compare whether there were significant differences in circulation, liver and kidney function, blood lipids, and delirium between the two groups.

### Analgesia and sedation protocol

Analgesia protocol: The analgesic drug was administered intravenously at a continuous rate of 0.02–0.15 mcg/kg of remifentanil (RF) per minute. The critical care pain observation tool (CPOT) score was maintained at 0. The detailed scoring criteria are presented in Supplementary Table 2.

Sedation protocol: In the experimental group, the sedative medication consisted of a continuous infusion of remimazolam besylate (Yichang Human-well Pharmaceutical Co., Ltd., China) at a dosage of 0.01–0.04 mg/kg/h. Given that there have been no experimental studies on the use of remimazolam for procedural sedation in the ICU, the dosages of remimazolam used in this clinical trial were derived from iterative adjustments made during the study. In the control group, propofol (Nhwa Pharmaceutical Co., Jiangsu, China) was administered intravenously, beginning with an initial loading dose of 1–2 mg/kg, followed by a continuous infusion of propofol at a rate of 1–4 mg/kg/h. The Richmond Agitation Sedation Scale (RASS) score ranged from − 2–0 (Table S3).

The administration of analgesic and sedative drugs was ceased for all patients between 08:30 and 09:00 a.m. daily until they were fully conscious. Patients were evaluated based on their ability to open their eyes and complete simple commands (e.g., shaking hands, extending two fingers). Once the standard was met, a weaning evaluation (spontaneous breathing test) was conducted. For patients unable to be weaned, analgesic and sedative treatment was resumed at half the original dosage, with the dosage being gradually adjusted to achieve the target level of sedation (RASS score of − 2 ~ 0). Sedation was discontinued once the weaning conditions were met.

### Outcomes

The primary outcome was the haemodynamic effects of remimazolam in mechanically ventilated patients with tracheal intubation, and the mean arterial pressure (MAP = DBP + 1/3(SBP‒DBP)) was used as the primary outcome measure. Secondary outcomes were assessed in terms of drug safety, adverse reactions, short-term patient prognosis, and the economic benefits of remimazolam. Indicators for drug safety include hepatotoxicity, nephrotoxicity, and allergic reactions. Adverse reactions were assessed by observing whether patients had abnormal blood lipids or developed delirium. Short-term prognosis was evaluated via indicators such as total mechanical ventilation time, time from ventilator use to tracheal intubation, 20-day in-hospital mortality, and ICU length of stay. The economic benefits of remimazolam were assessed based on sedation costs and average daily drug doses.

### Statistical analysis

Numerical data are presented herein as either the mean ± standard deviation if they were normally distributed or as the median (interquartile range) if they were not normally distributed. If the samples had equal variances and followed a normal distribution, the independent-sample T test was employed. Otherwise, the Mann‒Whitney U test or data transformation was used to fulfil the requirements for analysis of variance. Categorical data are presented as frequencies or percentages. The chi-square test was used to compare two groups, with a significance level of *P* < 0.05 indicating statistical significance. In addition, we also performed a multiplicity analysis before unbinding.

## Results

We conducted a statistical analysis of the baseline characteristics of the patients. The median age of the patients was 65.79 ± 13.59 years, with 48 males (60.0%), and the average BMI was 22.42 ± 2.73 kg/m^2^. There were no significant differences in demographic or baseline characteristics between the two groups regarding age, sex, BMI, or other demographic factors (Table 1).

**Table 1:** Patient characteristics

	Propofol group	Remimazolam group	All	*P*	χ^*2*^
	(n = 40)	(n = 40)	(n = 80)		
Age (years)	65.75 ± 13.20	65.82 ± 14.13	65.79 ± 13.59	0.878	
Sex (male/female)	25/15	24/17	48/32	0.820	
BMI (kg/m^2^)	22.31 ± 2.23	22.54 ± 3.18	22.42 ± 2.73	0.900	
MAP (mmHg)	92.19 ± 18.94	94.09 ± 18.51	93.14 ± 18.63	0.615	
SOFA score	6.68 ± 2.73	7.83 ± 3.15	7.25 ± 2.99	0.155	
APACHE II score	22.98 ± 6.58	23.70 ± 7.45	23.34 ± 7.00	0.646	
*Basic diseases (%)*					
Hypertension	19(47.5)	24(60.0)	43(53.8)	0.370	1.257
Diabetes	10(25.0)	10(25.0)	20(25.0)	1.000	0.000
Chronic heart disease	7(17.5)	3(7.5)	10(12.5)	0.311	1.829
Central disease	11(27.5)	14(35.0)	25(31.3)	0.630	0.524
*Causes of mechanical ventilation (%)*					
Cardiogenic	6(15.0)	7(17.5)	13(16.3)	1.000	0.092
Pulmonary origin	14(35.0)	12(30.0)	26(32.5)	0.812	0.228
Other	20(50.0)	21(52.5)	41(51.2)	1.000	0.050
*Laboratory examination*					
PCT (ng/ml)	6.68 ± 11.42	8.62 ± 22.25	7.71 ± 17.94	0.108	
WBC (× 10^9^/L)	11.16 ± 5.87	12.66 ± 6.77	11.91 ± 6.34	0.513	
HB (g/L)	102.50 ± 31.52	114.90 ± 31.50	109.20 ± 32.03	0.061	
PLT (× 10^9^/L)	171.29 ± 87.74	186.88 ± 110.03	179.08 ± 99.19	0.668	
CRP (mg/L)	160.25 ± 188.20	148.46 ± 193.03	154.84 ± 188.92	0.496	
Lac (mmol/L)	3.25 ± 3.80	3.95 ± 3.72	3.60 ± 3.76	0.200	
Shock (%)	7(17.5)	12(30.0)	19(23.8)	0.293	1.726
Vasoactive drugs	5(12.5)	11(27.5)	16(20.0)	0.161	2.813

The data are presented as counts (%), means (SDs), or medians (interquartile ranges)

BMI, body mass index; APACHE II, Acute Physiology Chronic Health Evaluation II; SOFA, Sequential Organ Failure Assessment; MAP, mean artery pressure; PCT, procalcitonin; WBC, white blood cell; NE%, neutrophil percentage; HB, haemoglobin*;* PLT, platelet; CRP, hypersensitive *C-*reactive protein; Lac, lactic acid

**Fig. 2:**
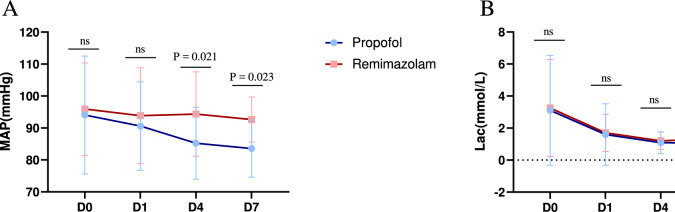
Comparison of the MAP (A) and Lac (B) between the remimazolam group and the propofol group. There were significant differences between the remimazolam group and the propofol group on Days 4 (*P* = 0.021) and 7 (*P* = 0.023). Abbreviations: MAP, artery pressure; Lac, lactic acid

We observed that patients who received procedural sedation with remimazolam had more stable haemodynamics and were less prone to hypotension. Statistical analysis revealed significant differences in the mean arterial pressure between the two groups on Days 4 and 7 (*P* = 0.021, control group vs. experimental group = 85.23 ± 11.24 vs. 94.36 ± 13.18, *P* = 0.023, 83.55 ± 8.94 vs. 92.66 ± 7.02) (Fig. 2). We also calculated the incidence of decreased MAP compared with previous levels in remimazolam and propofol groups. The statistical results showed that on Day 4, the incidence of MAP decrease was 77.8% in the propofol group and 25.9% in the remimazolam group (P < 0.01). On Day 7, the incidence of MAP decrease was 78.6% in the propofol group and 27.3% in the remimazolam group (P = 0.012). We found that the rate of MAP reduction from the day of admission to day 4 was 92.6% in the propofol group and 66.7% in the remimazolam group (P = 0.039). The rate of MAP decline from the day of admission to the 7th day was 92.9% in the propofol group and 45.5% in the remimazolam group (P = 0.021) (Table S4).

**Fig. 3:**
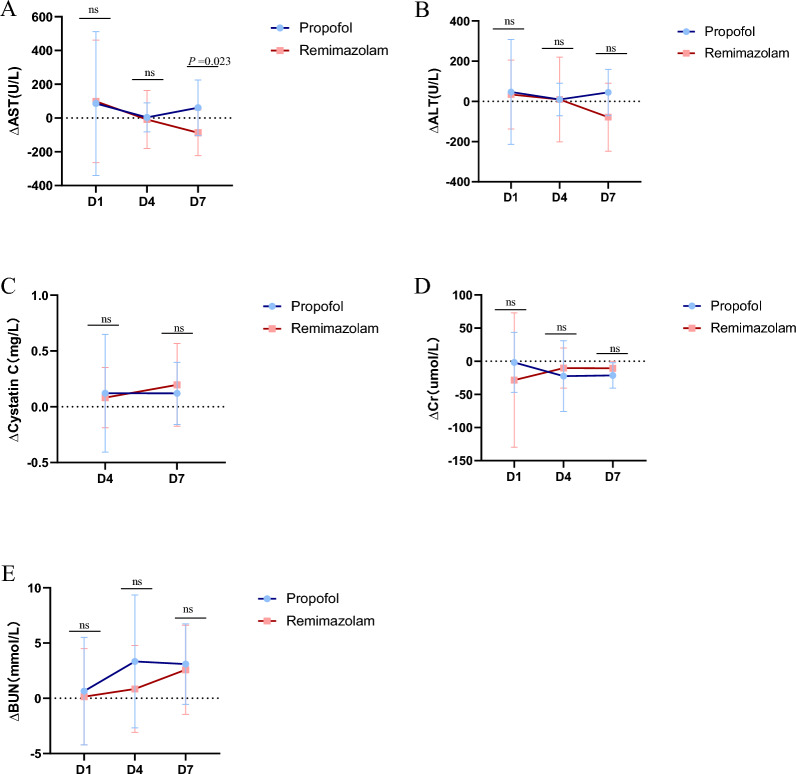
The ratio of liver(A-B) and renal(C-E) function between the remimazolam group and the propofol group. (B)The remimazolam group had a significantly lower ΔAST on day 7 (*P* = 0.023). Abbreviations: AST, aspartate transaminase; ALT, alanine aminotransferase; Δ Difference between Day 1, Day 4, Day 7, and the day of enrolment

Additionally, we statistically analysed liver and kidney function on Days 0, 1, 4, and 7 and reported that the ∆AST value was significantly lower in the remimazolam group than in the propofol group on Day 7 (*P* = 0.023,87.25 ± 135.56 vs. − 61.00 ± 164.51). There were no significant differences on Days 1 and 4 (*P* > 0.05), and no notable differences were observed in the ∆ALT or kidney function between the two groups (*P* > 0.05) (Fig. 3).

During the study, no allergic reactions were observed in either group.

**Fig. 4:**
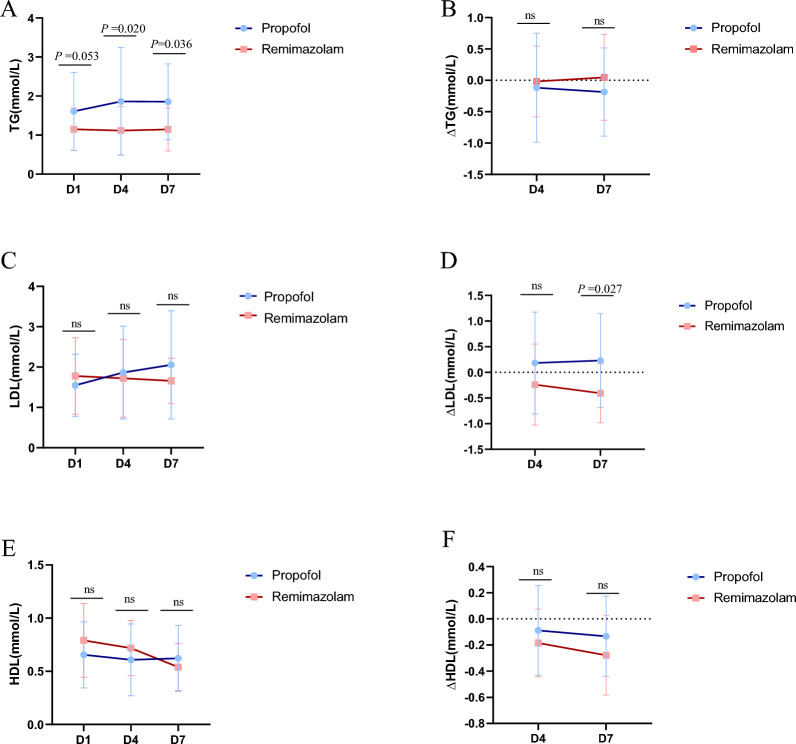
Ratio of blood lipids including TG（A-B）LDL（C-D）HDL(E-F) in the remimazolam group and propofol group. (A)There were statistically significant differences in TG levels between the remimazolam group and the propofol group on the 4th and 7th days (*P* = 0.020,* P* = 0.036), and (D)there were also significant differences in ΔLDL on the 7th day (*P* = 0.027). Abbreviations: TG, triglyceride; LDL, low-density lipoprotein; HDL, high-density lipoprotein; D4Δ, difference between the 4th day and the 1st day; D7Δ, difference between the 7th day and the 1st day

We subsequently conducted statistical analyses on blood samples from patients on Days 1, 4, and 7 and detected significant differences in triglyceride (TG) levels on Days 4 and 7 (*P* = 0.020, control group vs. experimental group = 1.86 ± 1.37 vs. 1.11 ± 0.62; *P* = 0.036, 1.85 ± 0.97 vs. 1.14 ± 0.55) and in the ∆LDL on Day 7 (*P* = 0.027, 0.23 ± 0.91 vs. −0.40 ± 0.57) (Fig. 4). Although no significant differences were observed in LDL, HDL, ∆TG, or ∆HDL (P > 0.05), 36 patients in the propofol group (90%, n = 40) and 34 (85%, n = 40) in the remimazolam group (Table 2) developed dyslipidaemia.

**Table 2:** The incidence of dyslipidaemia and delirium in the remimazolam group and propofol group

	Incidence [%(n/N)]	χ^*2*^	*P*
Dyslipidaemia			
Propofol group (n = 40)	90.0 (36/40)	0.499	0.737
Remimazolam group(n = 40)	85.0 (34/40)		
Delirium			
Propofol group(n = 40)	55.0 (22/40)	0.012	0.022
Remimazolam group(n = 40)	27.5 (11/40)		

Owing to the frequent occurrence of delirium in ICU patients, we also assessed the incidence of delirium in the included patients via the CAM-ICU rating scale. In the remimazolam group, 11 patients experienced delirium, resulting in an incidence rate of 27.5% (n = 40), whereas in the propofol group, 22 patients experienced delirium, with an incidence rate of 55% (n = 40). The incidence of delirium was significantly lower in the remimazolam group than in the propofol group (*P* = 0.022) (Table 2).

There were no differences in mechanical ventilation duration, hospital stay, time from extubation to decannulation, or 20-day mortality.

**Fig. 5:**
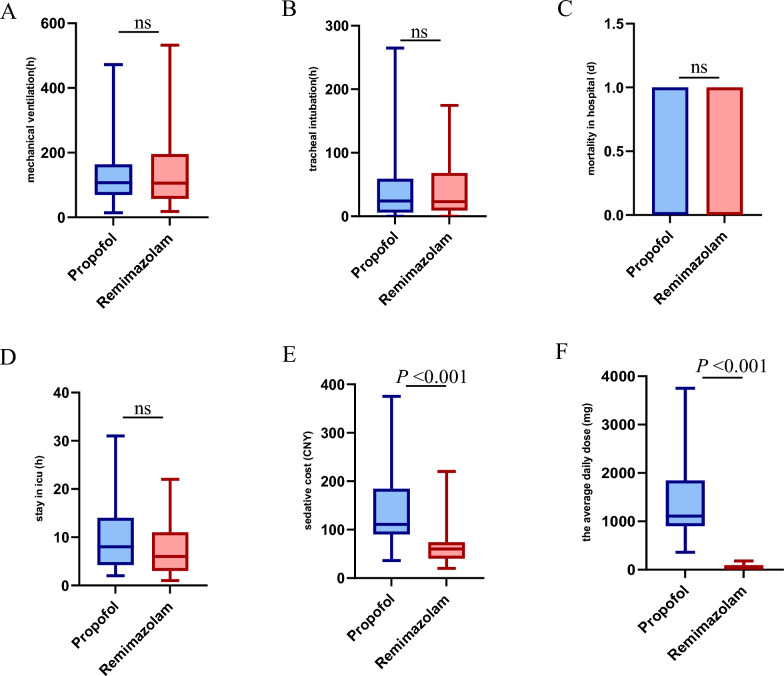
**A**: Total mechanical ventilation time;** B**: time from ventilator to tracheal intubation;** C**: 20-day mortality in the hospital;** D**: length of stay in the ICU;** E**: sedative cost;** F**: average daily dose. (E-F) Compared with the propofol group, the remimazolam group presented significantly lower average sedation costs and daily drug doses (P < 0.001, P < 0.001). There was no significant difference in the other variables

However, there was a significant reduction in the dosage of sedatives (*P* < 0.001, control group vs. experimental group = 1346.04 ± 717.82 vs. 53.86 ± 31.34) and sedation costs (*P* < 0.001) for patients who received remimazolam (Fig. 5).

## Discussion

In this study, we investigated critically ill intubated patients receiving procedural sedation with remimazolam and reported that remimazolam performed better than propofol in maintaining haemodynamic stability, particularly mean arterial pressure (MAP), making it a suitable option for critically ill patients with haemodynamic instability.

During the study, we discovered many interesting findings. First, sedative drugs can easily cause a decrease in blood pressure, and critically ill patients often have unstable haemodynamics. Therefore, the choice of sedative drug needs to be carefully considered. In the study by Tang et al. on deep sedation with remimazolam in critically ill patients, the incidence of hypotension with remimazolam was lower than that with propofol [13]. In our study, we discovered that, at the same level of sedation, the incidence of hypotension in patients undergoing procedural sedation with remimazolam was lower than that in the propofol group. Patients in the propofol group were more likely to develop hypotension, which is consistent with the known vasodilation and myocardial depression effects of propofol. In contrast, the MAP in the remimazolam group remained stable, whereas the MAP in the propofol group gradually decreased during treatment. On this basis, we further analyzed the incidence of MAP reduction compared to pre-admission levels in the two groups. It was found that the incidence of MAP reduction in the remimazolam group was significantly lower than that in the propofol group at all time points (D4, D7) and throughout the entire hospitalization period (D0 to D4, D0 to D7). This indicates that using remimazolam for procedural sedation during hospitalization has a smaller impact on patients' mean arterial pressure and better maintains hemodynamic stability. Although there was no statistically significant difference in lactate levels between the two groups, lactate levels in both groups tended to decrease over time, indicating improved haemodynamics and enhanced tissue oxygenation. These findings also suggest that both drugs have similar effects on metabolic burden and tissue perfusion, neither significantly increasing metabolic stress nor causing tissue hypoxia. These findings suggest that remimazolam has no inhibitory effects on the myocardium and has minimal impact on heart rate, making it potentially more suitable than traditional sedatives for intubated patients with haemodynamic instability. Its sedative applications appear to be broader [14].

Moreover, the conventional sedative drug propofol is metabolised mainly by glucuronidation in intrahepatic microsomes, and some is metabolised by glucuronidation in renal microsomes; this process is catalysed by the uridine diphosphate glucuronosyltransferase (UGT) isoform [15, 16]. It is ultimately excreted through the kidneys in the form of a water-soluble conjugate [17, 18]. The relevant literature indicates that remimazolam is metabolised primarily by tissue carboxylesterases. Its metabolites are inactive; however, the specific role of these tissue esterases in its metabolism is not yet fully understood and requires further experimental investigation [19].

In our study, patients in the remimazolam group showed more significant improvement in liver function, particularly on Day 7, suggesting that remimazolam may have a lesser impact on liver function. Additionally, as shown in the Fig. 3, the ΔAST levels in remimazolam patients tended to decrease, indicating that remimazolam may have potential hepatoprotective effects. The changes in ALT levels were not significant, suggesting that the effects of remimazolam and propofol on hepatocyte injury may be similar. Using remimazolam for sedation can alleviate inflammation in patients with liver injury and reduce oxidative stress and cell apoptosis [20]. Combining our data with the relevant literature, we hypothesise that remimazolam is suitable for procedural sedation in patients with impaired liver function. However, to ensure patient safety, our study did not conduct further verification experiments.

Next, cystatin C is a sensitive indicator of kidney function, and the lack of significant differences suggests that remimazolam and propofol have similar effects on renal function. Additionally, the nonsignificant changes in creatinine and blood urea nitrogen levels further support that the impact of remimazolam on renal function is comparable to that of propofol. Therefore, whether remimazolam has a superior effect on kidney function compared with that of propofol requires validation through larger-scale studies.

Furthermore, allergic reactions to remimazolam are relatively rare in current clinical research and practice. However, as a novel benzodiazepine sedative, its potential allergic reactions still warrant attention. Allergic reactions to remimazolam manifest primarily as mild skin reactions or localised symptoms, with severe allergic reactions being extremely rare [21]. Although no allergic reactions were observed in the patients included in our experimental study, it is essential to strengthen the screening of patients’ allergy history and closely monitor the first drug administration in clinical practice to ensure drug safety.

High levels of fat in the blood, known as hyperlipidaemia, are known risk factors for heart- and brain-related diseases. Critically ill patients with hyperlipidaemia are particularly at risk for these types of health issues. On Day 4 and Day 7, TG levels in the remimazolam group were significantly lower than those in the propofol group, indicating that remimazolam is superior to propofol in reducing triglyceride levels and may have a positive impact on lipid metabolism. Although the TG levels were significantly different, the changes (ΔTG) were not significant, possibly due to individual differences or observation time points. The effects of remimazolam and propofol on LDL levels were similar; however, the ΔLDL in the remimazolam group was significantly lower than that in the propofol group, suggesting that remimazolam may have a greater advantage in reducing LDL levels. While neither drug had a significant effect on HDL or ΔHDL levels, overall, remimazolam appears to be more suitable for critically ill patients with hyperlipidaemia, particularly those requiring improved triglyceride levels. Moreover, the metabolic properties of remimazolam may reduce the burden on lipid metabolism, contributing to better overall metabolic management in critically ill patients. Analysis of the incidence of dyslipidaemia revealed that the remimazolam group had a slightly lower incidence than did the propofol group, although the difference was not statistically significant. While the overall incidence of dyslipidaemia did not differ significantly between the two groups, remimazolam demonstrated a more favourable regulatory trend. For patients at risk of hyperlipidaemia or lipid metabolism disorders, remimazolam may be a more appropriate choice for sedation.

Delirium is a common symptom in ICU patients, and its occurrence can prolong hospital stays, increase medical costs, and increase the risk of death [22–24]. Therefore, delirium prevention in critically ill patients is highly necessary. In recent years, numerous scholars have conducted extensive research on the pharmacological prevention and treatment of delirium, including the use of antipsychotics, dexmedetomidine, propofol, and benzodiazepines [25–27]. Benzodiazepines are only indicated for delirium prevention in specific circumstances, such as abstinence from benzodiazepines themselves or alcohol [28, 29].

In our study, remimazolam significantly reduced the incidence of delirium, indicating that while its sedative effects were maintained, it had a lesser effect on the nervous system. This characteristic helps lower the risk of delirium in ICU patients and improves patient outcomes. We believe that, compared with propofol, remimazolam may be a superior sedative option, particularly for critically ill patients or those at high risk of delirium. However, there is currently limited research on the use of remimazolam for procedural sedation in critically ill patients, and more clinical data are needed to support its efficacy in preventing delirium.

In addition, we found that the sedation drug costs in the remimazolam group were lower than those in the control group, indicating the economic advantage of remimazolam in reducing medical expenses for ICU patients. Moreover, remimazolam requires a smaller dosage, suggesting that it may be more efficient in achieving sedation effects while potentially reducing the risk of drug-related side effects. There were no significant differences between remimazolam and propofol in terms of mechanical ventilation duration, tracheal intubation time, in-hospital mortality, or ICU length of stay. These findings indicate that both drugs have similar effects on short-term physiological recovery and prognosis. Given the lack of significant differences in short-term prognostic indicators (e.g., mortality and ICU length of stay), the economic benefits and efficiency of remimazolam provide potential advantages. Remimazolam may be more suitable for critically ill patients who aim to reduce sedation costs or minimise drug dosages. Overall, as a novel ultrashort-acting benzodiazepine sedative, remimazolam has advantages such as fewer side effects, rapid metabolism, and lower dosing, making its application range broader than that of propofol. It is safe for procedural sedation in critically ill patients.

Some limitations of our study should also be considered. First, owing to the peculiarities of the appearance of the sedative drug, we could not carry out complete blinding. Second, owing to the small number of experimental subjects, we did not conduct an in-depth study on another side effect of remimazolam—respiratory depression—in patients undergoing procedural sedation for intubation, and we did not obtain relevant experimental results. Additionally, all participants in this study were screened and enrolled after ICU admission without undergoing a baseline delirium assessment or cognitive function assessment, so we cannot rule out potential bias from an imbalance in baseline conditions prior to enrolment. Finally, owing to the lack of research on the use of remimazolam for procedural sedation in critically ill patients, substantial clinical trials are still needed to confirm the sedation effects of remimazolam in ICU patients and to obtain more supporting clinical data.

## Conclusions

In summary, remimazolam is a novel benzodiazepine that has demonstrated promising applications in general anaesthesia and procedural sedation. However, its use in ICU sedation is still in the early stages of research. Current evidence suggests that remimazolam is a safe sedative that is particularly well suited for patients with haemodynamic instability. Large sample-size randomised clinical trials are warranted.

## Supplementary Information

Additional file 1.

## Data Availability

All data associated with the present study are available in the main text or supplementary materials.

## Abbreviations

ICU: Intensive care unit
CAM-ICU: Confusion assessment method of intensive care unit
CPOT: Critical care pain observation tool
RASS: Richmond agitation sedation scale
LAC: Lactate
TG: Triglyceride
LDL: Low-density lipoprotein
HDL: High-density lipoprotein
AST: Aspartate aminotransferase
ALT: Alanine aminotransferase
RF: Remifentanil
MAP: Mean arterial pressure
WBC: White blood cell
HB: Haemoglobin
PCT: Procalcitonin
NE%: Neutrophilic granulocyte percentage
CRP: C-reactive protein
APACHE II: Acute physiology and chronic health evaluation II
SOFA: Sequential organ failure assessment
BMI: Body mass index
CRRT: Continuous renal replacement therapy
