# Supplementary material for: Procedural sedative effect of remimazolam in ICU patients on invasive mechanical ventilation: a randomised, prospective study
Source: Ann Intensive Care. 2025 Jan 14;15:8. doi: 10.1186/s13613-025-01431-5 (PMC11732822; doi:10.1186/s13613-025-01431-5)
Supplement: Supplementary file 1 — Additional file 1. [file 13613_2025_1431_MOESM1_ESM.docx]

**Table** **S1.CAM-ICU Scoring Table**

The following is the CAM-ICU scoring table used for rapid assessment of delirium in ICU patients:

| Feature | Assessment Content | Criteria | Result |
| --- | --- | --- | --- |
| Feature 1: Acute Onset and Fluctuating Course | 1. Is there an acute change in mental status?P2. Is there fluctuation in symptoms (e.g., worsening or improvement over the day)? | Yes: Feature 1 PositivePNo: Feature 1 Negative | Positive/Negative |
| Feature 2: Inattention | Assess through attention tests (e.g., identifying "A" among a sequence of letters or completing simple tasks):PCan the patient follow instructions?PIs there a noticeable attention deficit? | Yes: Feature 2 PositivePNo: Feature 2 Negative | Positive/Negative |
| Feature 3: Altered Level of Consciousness | Assess using RASS (Richmond Agitation-Sedation Scale):PIs the RASS score 0 (alert and calm)? | Yes: Feature 3 PositivePNo: Feature 3 Negative | Positive/Negative |
| Feature 4: Disorganized Thinking | Assess through logical questions:PFor example: "Is a stone heavier than a leaf?"PIs the patient’s thinking disorganized or illogical? | Yes: Feature 4 PositivePNo: Feature 4 Negative | Positive/Negative |

**PDiagnostic Criteria:**

Delirium Positive:PThe following conditions must be met:P1. Feature 1 Positive (Acute Onset and Fluctuating Course).P2. Feature 2 Positive (Inattention).P3. Either Feature 3 (Altered Level of Consciousness) or Feature 4 (Disorganized Thinking) Positive.PPDelirium Negative:PThe above conditions are not met.

**Table S2. CPOT Scoring Table**

The following is the CPOT (Critical Care Pain Observation Tool) scoring table, used for assessing pain in ICU patients who cannot self-report:

| Category | Description | Score | Criteria |
| --- | --- | --- | --- |
| Facial Expression | Relaxed, neutral expressionPTense facial muscles (e.g., frowning, grimacing)PGrimacing or severe frowning | 0P1P2 | Relaxed: 0PTense: 1PGrimacing: 2 |
| Body Movements | No movement or normal positionPProtective movements, slow or cautiousPRestlessness, resistance to care, withdrawal of affected body part | 0P1P2 | No movement: 0PProtective movement: 1PRestless or resisting: 2 |
| Muscle Tension (evaluated by passive flexion and extension of upper limbs) | RelaxedPIncreased tone, tense and rigidPVery tense or rigid | 0P1P2 | Relaxed: 0PIncreased tone: 1PVery tense or rigid: 2 |
| Compliance with Ventilation (intubated patients) | Tolerating ventilator or vocalizationPCoughing or gagging on ventilator, unable to calm downPFighting the ventilator | 0P1P2 | Tolerating: 0PCoughing: 1PFighting: 2 |
| Vocalization (extubated patients) | No vocalizationPSighing, moaningPCrying out, screaming | 0P1P2 | No vocalization: 0PMoaning: 1PScreaming: 2 |

**PScoring Instructions:**

The CPOT score is calculated by summing the scores of all categories. Scores range from 0 (no pain) to 8 (severe pain). Interpretation:P- 0: No painP- 1-2: Mild painP- 3-4: Moderate painP- ≥5: Severe painPPFor intubated patients, use the "Compliance with Ventilation" category. For extubated patients, use the "Vocalization" category.

**Table S3. RASS Scoring Table**

The following is the RASS (Richmond Agitation-Sedation Scale) scoring table, used to assess agitation and sedation levels in ICU patients:

| Score | Description |
| --- | --- |
| +4 | Combative: Overtly combative, violent, immediate danger to staff. |
| +3 | Very Agitated: Pulls or removes tubes or catheters; aggressive. |
| +2 | Agitated: Frequent non-purposeful movement, fights ventilator. |
| +1 | Restless: Anxious but movements not aggressive or vigorous. |
| 0 | Alert and Calm: Spontaneously pays attention to caregiver. |
| -1 | Drowsy: Not fully alert, but has sustained awakening (eye-opening/eye contact) to voice (≥10 seconds). |
| -2 | Light Sedation: Briefly awakens with eye contact to voice (<10 seconds). |
| -3 | Moderate Sedation: Movement or eye opening to voice (but no eye contact). |
| -4 | Deep Sedation: No response to voice, but movement or eye opening to physical stimulation. |
| -5 | Unarousable: No response to voice or physical stimulation. |

**PScoring Instructions:**

The RASS score ranges from +4 (most agitated) to -5 (most sedated). It is used to assess the level of sedation or agitation in ICU patients to guide sedation management.

|  | **Incidence (%)** | ***P*** |
| --- | --- | --- |
| **D_4_** |  |  |
| Propofol group | **77.8** | ＜0.01 |
| Remimazolam group | **25.9** |  |
| **D_7_** |  |  |
| Propofol group | **78.6** | 0.012 |
| Remimazolam group | **27.3** |  |
| **D_0_→D_4_** |  |  |
| Propofol group | **92.6** | 0.039 |
| Remimazolam group | **66.7** |  |
| **D_0_→D_7_** |  |  |
| Propofol group | **92.9** | 0.021 |
| Remimazolam group | **45.5** |  |

**Table S4. Incidence of decreased MAP compared with previous levels in remimazolam and propofol groups.**

D0: The day of admission; D4: The 4th day after admission; D7: The 7th day after admission.

D4, D7: The rate of decrease in MAP compared to D0. D0→D4, D0→D7: The rate of decrease in MAP from D0 to D4 and from D0 to D7, respectively.
